# Racial differences in the major clinical symptom domains of bipolar disorder

**DOI:** 10.1186/s40345-023-00299-3

**Published:** 2023-05-11

**Authors:** Kevin Li, Erica Richards, Fernando S. Goes

**Affiliations:** 1 Department of Psychiatry and Behavioral Science, Johns Hopkins University School of Medicine, 550 N. Broadway, Suite 204, Baltimore, MD 21205 USA; 2 Department of Mental Health, Johns Hopkins Bloomberg School of Public Health, Baltimore, MD USA

**Keywords:** Bipolar disorder, Racial disparity, Sleep disturbance, Insomnia, Psychosis

## Abstract

**Background:**

Across clinical settings, black individuals are disproportionately less likely to be diagnosed with bipolar disorder compared to schizophrenia, a traditionally more severe and chronic disorder with lower expectations for remission. The causes of this disparity are likely multifactorial, ranging from the effects of implicit bias, to developmental and lifelong effects of structural racism, to differing cultural manifestations of psychiatric symptoms and distress. While prior studies examining differences have found a greater preponderance of specific psychotic symptoms (such as persecutory delusions and hallucinations) and a more dysphoric/mixed mania presentation in Black individuals, these studies have been limited by a lack of systematic phenotypic assessment and small sample sizes. In the current report, we have combined data from two large multi-ethnic studies of bipolar disorder with comparable semi-structured interviews to investigate differences in symptoms presentation across the major clinical symptom domains of bipolar disorder.

**Results:**

In the combined meta-analysis, there were 4423 patients diagnosed with bipolar disorder type I, including 775 of self-reported as Black race. When symptom presentations were compared in Black versus White individuals, differences were found across all the major clinical symptom domains of bipolar disorder. Psychotic symptoms, particularly persecutory hallucinations and both persecutory and mood-incongruent delusions, were more prevalent in Black individuals with bipolar disorder type I (ORs = 1.26 to 2.45). In contrast, Black individuals endorsed fewer prototypical manic symptoms, with a notably decreased likelihood of endorsing abnormally elevated mood (OR = 0.44). Within depression associated symptoms, we found similar rates of mood or cognitive related mood symptoms but higher rates of decreased appetite (OR = 1.32) and weight loss (OR = 1.40), as well as increased endorsement of initial, middle, and early-morning insomnia (ORs = 1.73 to 1.82). Concurrently, we found that black individuals with BP-1 were much less likely to be treated with mood stabilizers, such as lithium (OR = 0.45), carbamazepine (OR = 0.37) and lamotrigine (OR = 0.34), and moderately more likely to be on antipsychotic medications (OR = 1.25).

**Conclusions:**

In two large studies spanning over a decade, we found highly consistent and enduring differences in symptoms across the major clinical symptom domains of bipolar disorder. These differences were marked by a greater burden of mood-incongruent psychotic symptoms, insomnia and irritability, and fewer prototypical symptoms of mania. While such symptoms warrant better recognition to reduce diagnostic disparities, they may also represent potential targets of treatment that can be addressed to mitigate persistent disparities in outcome.

**Supplementary Information:**

The online version contains supplementary material available at 10.1186/s40345-023-00299-3.

## Introduction

Racial disparities in outcomes are seen across most  medical disorders (Bailey et al. 2017) In psychiatry, significant research related to health disparities has focused on elevated rates of schizophrenia in Black minority populations compared to other races (Olbert et al. 2018; Heun-Johnson et al. 2021). However, disparities also manifest across other psychiatric phenotypes such as bipolar (Akinhanmi et al. 2018) and major depressive disorders (Flores et al. 2021). In contrast to schizophrenia, the rates of mood disorders in nationwide surveys (Flores et al. 2021; Jackson et al. 2006) are ostensibly lower in Black individuals, despite evidence that underrepresented minority populations may suffer greater chronicity and severity of illness (Flores et al. 2021). This may be particularly relevant for bipolar disorder where differences in diagnostic thresholds may lead to an under-diagnosis of the illness, with attribution of symptoms to other psychiatric diagnoses such as schizophrenia or major depression that may ultimately lead to suboptimal and possibly harmful treatment. While epidemiological studies show few significant differences in rates of bipolar disorder across race and ethnicities, (Blanco et al. 2017; Merikangas et al. 2011) they are limited by a small number of cases and are likely underpowered to detect significant differences. Clinical studies, on the other hand, consists of much larger number of cases, but also reflect the effects of cultural and socioeconomic factors that may result in differential access and acceptance of mental health care. While such factors are often considered as confounds in causal analyses, they are relevant for our understanding of the causes of health care disparities, and may represent primary drivers of disparities in health care outcomes (Gee and Ford 2011).

Beginning in the 1970s and throughout the next three decades, several reports (Simon 1973; Bell and Mehta 1980; Delbello et al. 2000; Blow et al. 2004) described an increased rate of the diagnosis of schizophrenia compared to bipolar disorder in Black individuals within various U.S. health care settings. What accounts for this disparity in diagnosis may reflect a complex mixture of differing risk factors, (Vassos et al. 2020) structural barriers, (Bailey et al. 2017) implicit biases in clinical decision making, (Jarvis 2012) and potentially, the presentation of an untreated illness of greater severity (Roukema et al. 2022). An additional consideration may be that because of differing risk factors and/or paths to clinical presentation, Black individuals may have less “prototypical” manifestations of bipolar disorder, adding further complexity to the clinical evaluation and diagnostic decision making process.

While prior studies have noted a greater preponderance of specific psychotic symptoms (such as persecutory delusions and hallucinations) and dysphoric/mixed symptoms during manic episodes in Black individuals, (Akinhanmi et al. 2020) they did not compare across the major domains of bipolar illness (manic, depressive and psychotic) and were limited by modest sample sizes. Here in this report, we therefore combine two large multi-ethnic studies of bipolar disorder (Smith et al. 2009; Pato et al. 2013) with comparable semi-structured interviews to comprehensively investigate differences in symptom presentation across the major clinical symptom domains of bipolar disorder.

## Methods

We combined two large studies with comparable diagnostic interviews to assess mood and psychosis symptoms in Bipolar Disorder type I: the National Institute of Mental Health (NIMH) Genetics Initiative (Smith et al. 2009) and the Genomic Psychiatry Cohort (GPC) (Pato et al. 2013). Both datasets were extracted from the NIMH Repository & Genomics Resource (https://www.nimhgenetics.org/). The NIMH dataset was restricted to the single patient case collection study by 11 sites in the U.S. from 2004 to 2009 and limited to subjects with Bipolar Disorder type I (N = 2580). The GPC cohort involved subjects collected from 2009 to 2014 across thirteen sites in the U.S. and abroad comprised of 1843 subjects with bipolar type I. Individuals in the NIMH study were interviewed with the Diagnostic Interview for Genetic Studies (DIGS) (Nurnberger et al. 1994), a semi-structured interview with high reliability (kappa 0.73–0.95) for major depression, bipolar disorder, and schizophrenia (Nurnberger et al. 1994). Individuals in GPC were interviewed the Diagnostic Interview for Psychosis and Affective Disorders (DI-PAD), (Pato et al. 2013) a semi-structured interview containing the Operational Criteria Checklist for Psychotic Illness and Affective Illness (OPCRIT) criteria (Azevedo et al. 1999) that similarly has been found to have high inter-rater reliability (kappa 0.81–0.83) for major mood and psychosis best-estimate lifetime diagnoses (Azevedo et al. 1999). Harmonization of both interviews was performed by extracting questions that specifically inquired about DSM-IV criteria for manic and depressive episodes as well as lifetime psychotic symptoms. Both interviews are derivatives based on the Present State Examination interview, (Wing et al. 1974) resulting in comparable mood and psychotic symptom questions for comparison.

We limited participants to those with bipolar disorder type I who self-identified as Black or White and extracted lifetime most severe episode symptoms for manic and depressive episodes. We additionally looked at lifetime history for psychotic symptoms and common comorbidities such as alcohol and drug abuse. The overall rates of missing symptom data after cleaning were 6.8% for NIMH and 3.2% for GPC. Imputation for missing symptom data was preformed using the multivariate imputation by chained equations (MICE) package in R. Multivariate logistic regression was performed in each study controlling for age, race, sex, alcohol abuse, and drug abuse. Subsequently, we performed a fixed-effects meta-analysis using the metafor package in R.

## Results

In the combined sample, there were 4423 individuals diagnosed with bipolar disorder type I, including 775 who self-identified as Black. Black individuals with bipolar disorder type I had a similar sex distribution (58.3% vs 59.7% of the sample being female, p = 0.5) as White individuals with bipolar disorder type I. There were comparable levels of lifetime alcohol abuse and dependence (49.9% vs 52.2%, p = 0.3), but higher levels of lifetime drug abuse and dependence comorbidity (53.0% vs 42.6%, p =  < 0.001) in Black individuals. The average age at entry was 43 years, with no difference seen between races. In both samples, we extracted and analyzed 9 manic symptoms, 15 depressive, and 14 psychosis related symptoms. Complete symptom report prevalence by race, odds ratios, and confidence intervals are available in Additional file 1: Table S1.

### Manic symptoms

**Fig. 1:**
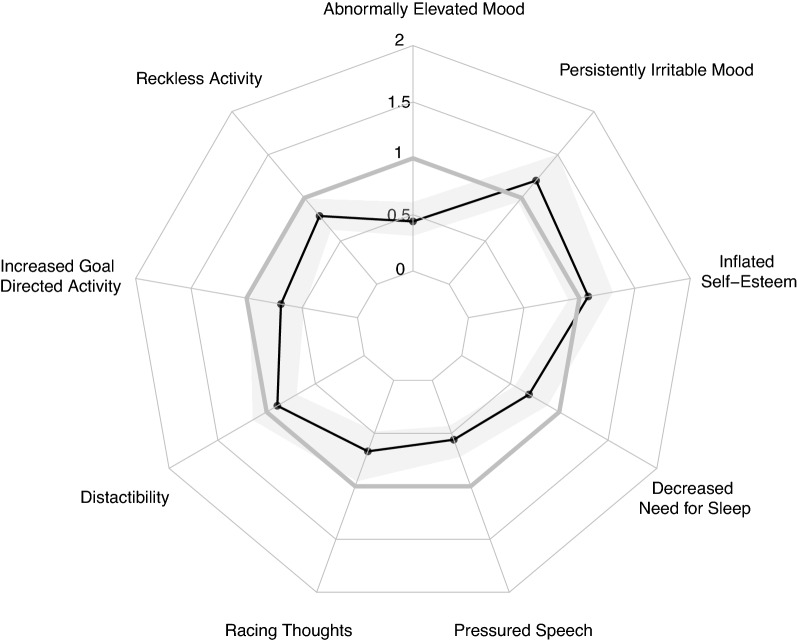
Meta-analytical estimates (Odds Ratios) comparing the prevalence of mania symptoms in Black versus White individuals with Bipolar I Disorder. Associations are corrected for age, sex and the prevalence of alcohol/substance abuse disorders at the level of each study prior to meta-analysis. Shaded band represents the 95% Confidence Interval surrounding the meta-analytic effect estimate

As shown in Fig. 1, Black individuals with mania were less likely to report most classically defined symptoms of mania, with lower endorsement of abnormally elevated mood (meta-OR = 0.44[95% CI = 0.31–0.61]), decreased need for sleep (meta-OR = 0.69[0.54–0.88]), pressured speech (meta-OR = 0.56[0.43–0.72]), racing thoughts (meta-OR = 0.67[0.48–0.96]), increased goal directed activity (meta-OR = 0.69[0.48–0.97]), and reckless activity (meta-OR = 0.79[0.63–0.99]).

### Depressive symptoms

**Fig. 2:**
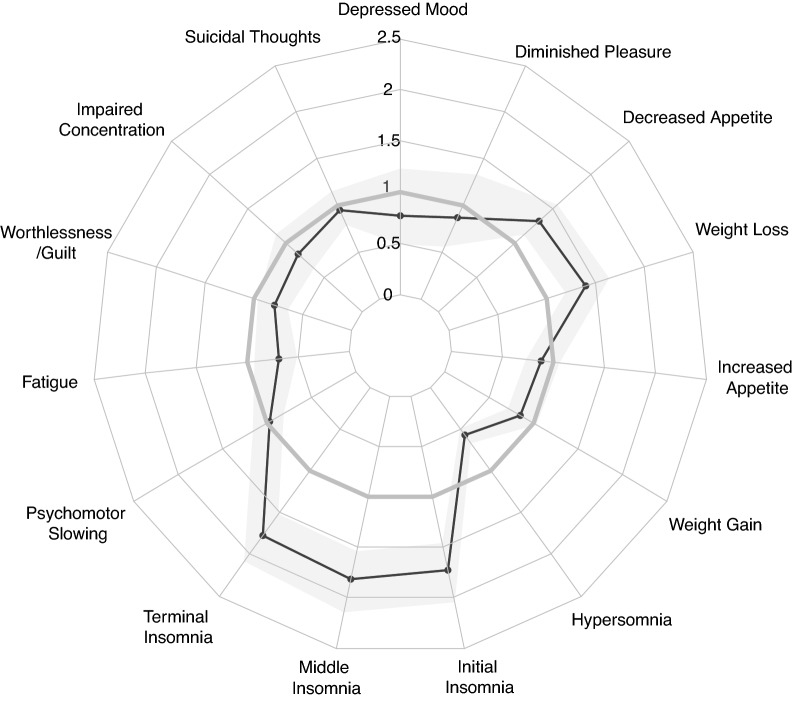
Meta-analytical estimates (Odds Ratios) comparing the prevalence of depression symptoms in Black versus White individuals with Bipolar I Disorder. Associations are corrected for age, sex and the prevalence of alcohol/substance abuse disorders at the level of each study prior to meta-analysis. Shaded band represents the 95% Confidence Interval surrounding the meta-analytic effect estimate

There were similarly robust differences in depression symptoms related to disturbed sleep (Fig. 2). Black individuals were more likely to report difficulty falling asleep, (“initial” insomnia meta-OR = 1.73[1.46–2.05]), difficulty remaining asleep (“middle” insomnia meta-OR = 1.82[1.54–2.15]), and greater likelihood of experiencing early morning awakening (“terminal” insomnia meta-OR = 1.78[1.52–2.09]). Conversely, hypersomnia was less commonly endorsed by Black individuals with bipolar disorder (meta-OR = 0.57[0.49–0.67]). Other depressive symptoms differentially reported by Black individuals included decreased appetite (meta-OR = 1.32[1.12–1.56]) and weight loss (meta-OR = 1.40[1.19–1.64]), as well as decreased tendency to report fatigue (meta-OR = 0.69[0.51–0.93]) and worthlessness/guilt (meta-OR = 0.79[0.65–0.96]) as core symptoms of their worst depression.

### Psychotic symptoms

**Fig. 3:**
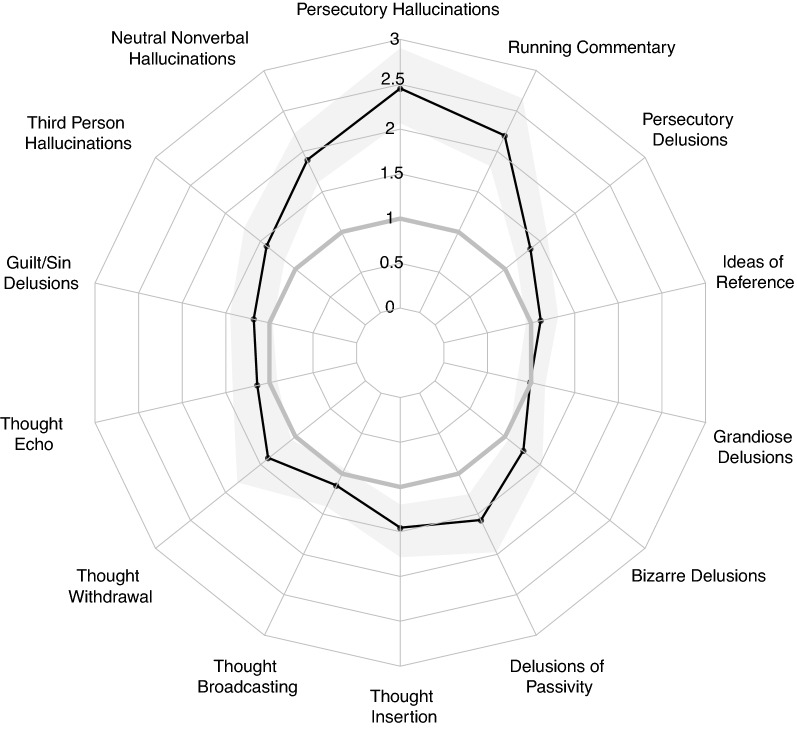
Meta-analytical estimates (Odds Ratios) comparing the prevalence of psychosis symptoms in Black versus White individuals with Bipolar I Disorder. Associations are corrected for age, sex and the prevalence of alcohol/substance abuse disorders at the level of each study prior to meta-analysis. Shaded band represents the 95% Confidence Interval surrounding the meta-analytic effect estimate

Psychotic symptoms were present in 71.4% of the sample, with a slight over-representation in Black individuals (OR = 1.15[1.03–1.30]). More specifically, Black individuals reported higher rates of several hallucinatory phenomena including neutral nonverbal hallucinations (meta-OR = 1.89[1.61–2.22]), persecutory hallucinations (meta-OR = 2.45[2.06–2.90]), running commentary hallucinations (meta = OR = 2.19[1.81–2.65]), and third person hallucinations (meta-OR = 1.41[1.15–1.73]) (Fig. 3). There were also increased mood-incongruent psychotic features, including persecutory delusions (meta-OR = 1.36[1.16–1.60]), delusions of passivity (meta-OR = 1.58[1.26–1.98]), thought insertion (meta-OR = 1.46[1.20–1.79]), and thought withdrawal (meta-OR = 1.39[1.06–1.83]).

### Pharmacotherapy

To explore potential treatment consequences of the differences in symptoms observed, we compared which medications were prescribed at the time of the diagnostic interview (Table 1). We found strong evidence of decreased use of conventional mood stabilizers in Black individuals, including lithium (meta-OR = 0.45[0.36–0.56]), lamotrigine (meta-OR = 0.34[0.26-0.44]), and carbamazepine (meta-OR = 0.37[0.21–0.65]). An exception was valproate, which showed comparable use rates across the two racial categories. Similarly, we found lower rates of antidepressant use (meta-OR = 0.82[0.70–0.96]) in Black individuals. On the other hand, antipsychotics were more likely to be used in Black individuals (meta-OR = 1.25[1.06–1.47]), although this pattern was largely driven by the NIMH study.

**Table 1:** Study specific prevalence of current medication use at time of enrollment in each perspective study followed by fixed effect meta-analysis comparing the prevalence of each medication class in Black versus White individuals with Bipolar I Disorder

Medication	NIMH Bipolar Initiative		Genomic Psychiatry Cohort		Combined	
	Black (N = 374)	White (N = 2206)	Black (N = 401)	White (N = 1442)	Meta OR (95% CI)	P-value
Lithium	51 (13.6%)	576 (26.1%)	42 (10.5%)	289 (20.0%)	**0.45 (0.35, 0.56)**	** < .001**
Valproate	89 (23.8%)	476 (21.6%)	90 (22.4%)	289 (20.0%)	**1.17 (0.97, 1.41)**	**.104**
Lamotrigine	31 (8.3%)	580 (26.3%)	31 (7.7%)	225 (15.6%)	**0.34 (0.26, 0.44)**	** < .001**
Carbamazepine	12 (3.2%)	172 (7.8%)	1 (0.2%)	41 (2.8%)	**0.37 (0.21, 0.65)**	** < .001**
Antipsychotic	242 (64.7%)	1186 (53.8%)	245 (61.1%)	870 (60.3%)	**1.25 (1.06, 1.47)**	**.007**
Antidepressant	174 (46.5%)	1224 (55.5%)	186 (46.4%)	687 (47.6%)	**0.82 (0.7, 0.96)**	**.014**

Associations are corrected for age, sex and the prevalence of alcohol/substance abuse disorders at the level of each study prior to meta-analysis

Bolded values reflect meta-analytic effect size estimates and significance

## Discussion

This study combined two large-scale collections of subjects with bipolar disorder from 2004 to 2014 to examine whether variations in symptom manifestation between the two most common racial categories in the United States, may be a contributing factor to the ongoing disparities in diagnostic rates and outcomes in individuals with bipolar disorder. Our study includes a substantially larger sample size compared to prior literature and utilized a systematic assessment of the major clinical symptom domains. Our research supports earlier studies that suggest Black individuals with bipolar disorder are more likely to experience psychotic symptoms (Akinhanmi et al. 2020). Additionally, our findings reveal significant racial disparities in both manic and depressive symptoms, which could result in a less typical manifestation of bipolar disorder and potentially lead to misdiagnosis. These results underscore the importance of considering race when assessing and treating individuals with bipolar disorder and call for further investigation into the underlying mechanisms driving these racial disparities.

Notably, we found robust differences in both manic and depressive sleep related disturbances in Black compared to White individuals with bipolar disorder type I. Sleep symptoms are less likely to be affected by provider interpretation and bias, and have been increasingly linked to adverse metabolic, cardiovascular and cognitive outcomes (Zangani et al. 2020). Poor sleep measures have been previously found to be more common in Black individuals in population based surveys (Carnethon et al. 2016; Kim et al. 2022) and may reflect an early and treatable manifestation of complex psychological distress in underrepresented minority populations. In subjects with bipolar disorder, impaired sleep and disrupted circadian function is a likely risk factor for illness recurrence and poor outcomes (Gold and Sylvia 2016). Failure to adequately address sleep symptoms in individuals with bipolar disorder could contribute to health outcome disparities, underscoring the need to prioritize effective treatment of these symptoms.

In regard to traditional manic symptoms, our findings show a notably lower endorsement of traditional manic symptoms such as abnormally elevated mood, pressured speech, and increased goal directed activity. This may reflect a greater likelihood of Black individuals to present with dysphoric or mixed rather than euphoric manic syndromes. Furthermore, the higher occurrence of hallucinatory experiences and mood-incongruent delusions, which are more prevalent during manic episodes than depressive episodes, (Goes et al. 2007) can lead to a presentation characterized by increased irritability, paranoia, and atypical psychotic symptoms.

The underlying factors contributing to the observed differences in symptomatology, diagnostic patterns, and treatment outcomes in bipolar disorder are likely to be multifaceted and complex (Anglin et al. 2021). Adverse risk factors that are more prevalent in minoritized communities may play a role, as well as inequities in the availability and quality of healthcare for a chronic and often recurring illness like bipolar disorder. A recent proposed framework for the study of disparities in mental health research noted various “domains of influence”, or risk factors, across levels of analyses that span individual, interpersonal, community and society levels (Alvidrez and Barksdale 2022). Such factors, usually considered under the rubric of social determinants of health (including disparities in income and education level, neighborhood safety, poor working conditions, structural and interpersonal discrimination) may themselves be causal factors contributing, for example, to an increased propensity for symptoms such as suspiciousness and paranoid delusions (Anglin and Lui 2021). Although the topic remains understudied, adverse social determinants of health and discrimination have also been linked to greater depressive symptoms (Yelton et al. 2022) and poorer quality of sleep (Johnson et al. 2019).

Our study has important strengths in terms of systematic assessment of symptoms and large sample size. However, there are a number of limitations that must also be considered. First, a significant limitation is the cross-sectional rather than a longitudinal design, which would have been more informative for studying differences in clinical outcome and treatment. As a proxy, we examined whether classes of medications used at the time of diagnosis were comparable and found significantly lower use of traditional mood stabilizers in Black compared to White individuals. Since mood stabilizers may be better tolerated and more effective in the prevention future bipolar episodes, (Goes 2023) this disparity in treatment may unfortunately contribute to a pattern of poorer outcomes in Black individuals with bipolar disorder. Secondly, our study was limited by the fact that the diagnostic evaluations were not conducted in a blinded manner with respect to race. As a result, it is difficult to determine whether the differences in symptom patterns that we observed were due to varying ratings by the diagnosticians or to differences in the way that patients reported their symptoms. Previous research has suggested that clinicians may place a disproportionate emphasis on the diagnostic relevance (Arnold et al. 2004) and severity of psychosis symptoms in Black individuals (Gara et al. 2012). Third, our study did not specifically ask subjects about the potential structural and interpersonal sources of discrimination that may be the primary drivers of health outcome disparities. Lastly, we acknowledge that our study’s overly simplistic classification of race as single categories is problematic. A more refined conceptualization of ancestry and ethnic identification would have likely been more informative for the characterization of the symptom domains and illness trajectories of the diverse individuals in our study.

## Conclusions

Our study highlights a persistent and highly consistent difference in phenotypic presentation across the major domains of bipolar illness that diverges from prototypical presentations of illness and may contribute to the diagnostic disparities seen between Black and White individuals. As such disparities are also correlated with a greater likelihood of suboptimal treatment, they represent prevalent and addressable sources of morbidity that warrant further attention.

## Supplementary Information

**Additional file 1:** **Table S1.** Complete symptom report prevalence as broken down by race, odds ratios/confidence intervals for each symptom in their respective study, and meta-analysis odds ratios/confidence intervals.

## Data Availability

Both datasets analyzed in this manuscript were extracted from and available on the NIMH Repository & Genomics Resource (https://www.nimhgenetics.org/).
